# Probabilistic Seismic Response Prediction of Three-Dimensional Structures Based on Bayesian Convolutional Neural Network

**DOI:** 10.3390/s22103775

**Published:** 2022-05-16

**Authors:** Tianyu Wang, Huile Li, Mohammad Noori, Ramin Ghiasi, Sin-Chi Kuok, Wael A. Altabey

**Affiliations:** 1 School of Civil Engineering, Southeast University, Nanjing 211189, China; ty_wang@seu.edu.cn; 2 International Institute of Urban Systems Engineering (IIUSE), Southeast University, Nanjing 211189, China; rghiasi.s@gmail.com (R.G.); wael.altabey@gmail.com (W.A.A.); 3 National and Local Joint Engineering Research Center for Intelligent Construction and Maintenance, Southeast University, Nanjing 211189, China; 4 Department of Mechanical Engineering, California Polytechnic State University, San Luis Obispo, CA 93407, USA; 5 State Key Laboratory of Internet of Things for Smart City, Guangdong-Hong Kong-Macau Joint Laboratory for Smart City, Department of Civil and Environmental Engineering, University of Macau, Macau, China; sckuok@um.edu.mo; 6 Department of Mechanical Engineering, Faculty of Engineering, Alexandria University, Alexandria 21544, Egypt

**Keywords:** seismic response, random vibration of structures, Bayesian deep learning, convolutional neural network

## Abstract

Seismic response prediction is a challenging problem and is significant in every stage during a structure’s life cycle. Deep neural network has proven to be an efficient tool in the response prediction of structures. However, a conventional neural network with deterministic parameters is unable to predict the random dynamic response of structures. In this paper, a deep Bayesian convolutional neural network is proposed to predict seismic response. The Bayes-backpropagation algorithm is applied to train the proposed Bayesian deep learning model. A numerical example of a three-dimensional building structure is utilized to validate the performance of the proposed model. The result shows that both acceleration and displacement responses can be predicted with a high level of accuracy by using the proposed method. The main statistical indices of prediction results agree closely with the results from finite element analysis. Furthermore, the influence of random parameters and the robustness of the proposed model are discussed.

## 1. Introduction

An earthquake is a natural disaster which causes catastrophic damage to structures [1,2]. To accurately compute the seismic dynamic response and evaluate the safety of structures, the randomness and uncertainty of structure parameters need to be taken into consideration [3,4,5,6,7,8]. To solve this problem, various methods have been proposed by researchers since the 1960s, such as the Monte-Carlo simulation [9,10], the orthogonal polynomial expansion method [11] and the probability density evolution method [12,13,14,15]. The analytic results using these methods are given in the form of probability distributions. However, for these methods, the random parameter probability distributions need to be obtained before the calculation. If the probability distributions are unknown or change, these methods are difficult to carry out.

Recently, the development of neural network and deep learning [16] has provided a powerful approach for seismic response prediction. Different types of neural networks or deep learning models are currently used to predict the dynamic response of structures. The earliest work utilizing this approach aimed at response analysis of the linear response of structures, without considering any inherent uncertainty or randomness, and the types of neural network models were limited to multi-layer feedforward neural network (multilayer perceptron) [17,18,19,20]. To solve the problem of structural nonlinearity, prior knowledge was introduced into the neural network [21,22,23] and several new types of neural networks were proposed. With improvement of computational efficiency, deep learning models, such as deep convolutional neural network [24,25,26], deep recurrent neural network [26,27,28,29] and deep convolutional recurrent neural network [30], were developed and applied for structural seismic response prediction. Currently, wind-induced and vehicle-induced dynamic response predictions have attracted attention. Wang et al. used the deep recurrent neural network in structural response prediction under wind load [31]. Li et al. utilized the deep long- and short-time memory models for dynamic response prediction of a train-bridge system [32]. The probability-based method is combined with neural network and provides a new tool for random seismic response analysis. Kim used deep convolution neural networks to predict the peak and characteristic value of structural dynamic response under random earthquake [33]. Li et al. used the Bayesian deep learning model to predict the response of vehicle-bridge systems [34]. Wang et al. applied the Bayesian neural network in the seismic response prediction of single-degree of freedom (SDOF) systems [35]. However, so far, research work on seismic response prediction of three-dimensional buildings, with random characteristics, has been very limited.

In this paper, a Bayesian deep learning model [36] with convolutional neural network is utilized to predict the seismic response of a 3D building with random characteristics. The Bayes backprop algorithm as a high efficiency training algorithm, used to train the deep learning model. The trained model is utilized to predict the random response of a 3D building and to obtain the probability distribution of both displacement and acceleration responses. Finally, the influence of random parameters and the robustness of the proposed Bayesian deep learning model are discussed. The organization of this paper is as follows: In Section 2, the Bayesian deep learning theory and Bayes backprop algorithm are introduced; Section 3 provides the details of the numerical example of a 3D building with random characteristics; Section 4 presents the training process of the proposed deep learning model and illustrates the response prediction results for the 3D building; In Section 5, the influence of structural random parameters and the robustness of the proposed model are discussed, and, finally, in Section 6, some conclusions are drawn.

## 2. Framework of Bayesian Deep Learning Model

### 2.1. Convolutional Neural Network

**Figure 1:**
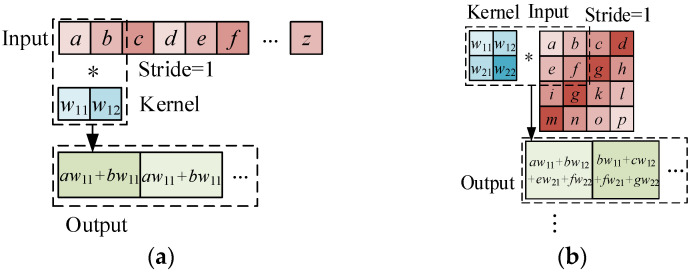
1-D and 2-D convolution operation: (**a**) 1-D convolution; (**b**) 2-D convolution.

Convolutional neural network (CNN) [37] is a typical deep learning model, which is mainly used to process grid data, such as voice, time series and images. Structural dynamic response data, as a type of time-series data, have been successfully predicted by using the CNN model in existing literature [24,27]. The basic concept is to use convolution operation, instead of fully connected layers, to extract features from the input data. This optimization realizes local connections and weight sharing which decrease the number of parameters. The definition of 1-D and 2-D convolutional operations in CNN can be expressed by Equations (1) and (2), respectively:

(1) $$o_{i,k}=(x*v{)}_{i,k}=\sum_{l,m}x_{(i-1)s+m,l}V_{m,l,k},$$

(2) $$O_{i,j,k}=(X*V{)}_{i,j,k}=\sum_{l,m,n}X_{(i-1)s+m,(j-1)s+n,l}V_{m,n,l,k},$$

where in Equation (1), **x** is the 1-D input, **v** is the convolutional kernel and **o** is the output. Similarly, in Equation (2), **X** is the input of the 2-D convolutional operation, and **V** and **O** are the corresponding kernel and output. The strides of convolutional operation, *s*, refers to how many data points are skipped between the two operations. The process of 1-D and 2-D convolution is expressed in Figure 1.

Pooling operation is another important operation used in CNN. The main function of pooling is to compress the data. There are mainly two types of pooling; mean pooling and maximum pooling. As its name implies, mean pooling outputs the mean value in the pooling region while maximum pooling outputs the maximum value.

**Figure 2:**
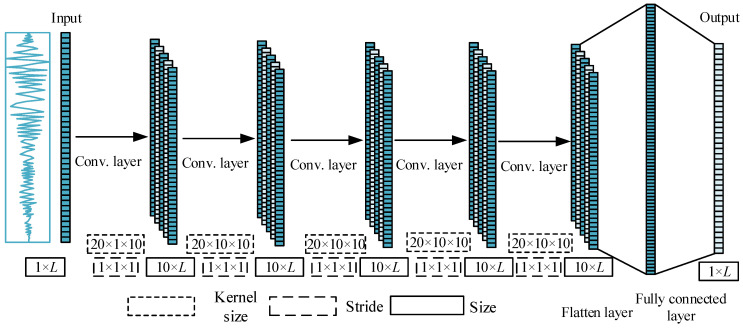
The architecture of the proposed CNN model for response prediction.

A 1-D CNN model is used to predict the seismic response of the building. The architecture of the proposed CNN model is described in Figure 2. The input is the earthquake acceleration, while the output is the displacement or the acceleration response. There are five hidden convolutional layers and one fully connected layer between input and output layers. For the convolutional layer, rectified linear unit (ReLU) is used as the activation function.

### 2.2. Training of Bayesian Deep Learning Model

When the deep learning model is built, the model parameters, such as weight matrix and bias vector, can be estimated by using the samples of input and output. This process is the so-called ‘model training’. From the perspective of probability theory, when the point estimation is utilized, the trained deep learning model has deterministic values. However, if Bayesian estimation is applied, the probability distribution can be obtained. In Bayesian deep learning, the variational inference is used to realize the Bayesian estimation.

(1) Variational inference

Let us consider a deep learning model with model parameter **W**. The set of training data includes M samples, such as: $S=\left\{\left(x_{1},y_{1}\right),\left(x_{2},y_{2}\right),\cdots ,\left(x_{M},y_{M}\right)\right\}$. According to the Bayes formula [38], the model parameters are estimated as:

(3) $$p\left(W|S\right)=\frac{p\left(S|W\right)p\left(W\right)}{p\left(S\right)}=\frac{p\left(S|W\right)p\left(W\right)}{\int_{W}p\left(S|W\right)p\left(W\right)dW}$$

where *p*(**W**) is the prior distribution, which is determined by assumption, prior knowledge or experience. The distribution of train samples is *p*(**S**), *p*(**S|W**) is the likelihood function and *p*(**W |S**) is the estimated distribution of model parameter **W**. Since *p*(**S**) is difficult to obtain, the Bayes formula cannot be used to obtain the parameter estimation directly. To solve this problem, a new distribution *q*(**W**) is introduced to approximate *p*(**θ|S**). The difference between *q*(**W**) and *p*(**W|S**) can be measured through the concept of Kullback-Leibler (KL) divergence [39]. KL divergence can be expressed as:

(4) $$\begin{array}{ll} D_{\mathrm{KL}}\left(q(\theta )||p(\theta |S)\right) & =\int_{W}q(W)\log\frac{q(W)}{p(W|S)}dW\\ & =\int_{\Theta }q(W)\log\frac{q(\theta )\int_{W}p(S|W)p(W)dW}{p(S|W)p(W)}dW\\ & =\int_{\Theta }q(W)p(S)dW-\int_{\Theta }q(W)\log\frac{p(S|W)p(\theta )}{q(W)}dW \end{array}$$

The aim of variational inference is to obtain the optimal *q*(**θ**) by minimizing KL divergence, or, in essence, maximizing the second term in the left as:

(5) $$q^{*}(W)=\underset{q(W)}{\arg\min}\mathrm{DKL}\left(q(W)||p(W|S)\right)=\underset{q(W)}{\arg\max}\int_{W}q(W)\log\frac{p(S|W)p(W)}{q(W)}dW,$$

In order to convert the variational problem to an optimization problem, the *q*(**W**) can be assumed to be a joint Gaussian distribution and every parameter *W**_i_*** in the parameter matrix **W** obeys an independent Gaussian distribution as:

(6) $$q(W)=N(W,\mu ,\sigma^{2})=\prod_{i}^{n_{W_{i}}}N(W_{i},\mu_{i},\sigma_{i}^{2}),$$

where **μ** and **σ** refer to the mean value matrix and the standard deviation. The optimal distribution *q*(**W**) can be obtained by finding the optimal value of parameter matrix **μ** and **σ** as:

(7) $$\begin{array}{ll} \;\mu^{*},\sigma^{*}= & \underset{\mu ,\sigma }{\mathrm{argmax}}\sum_{k=1}^{n_{\theta }}E_{q(\sigma_{k}\varepsilon_{k}+u_{k})}\left[\log(p\left(\sigma_{k}\varepsilon_{k}+u_{k}\right))\right]\\ & +\sum_{k=1}^{n_{\theta }}E_{q_{k}\left(\sigma_{k}\varepsilon_{k}+u_{k}\right)}\left[\log(q_{k}\left(\sigma_{k}\varepsilon_{k}+u_{k}\right))\right]\\ & +\frac{N_{B}}{N}\sum_{j=1}^{N/N_{B}}\frac{N}{N_{B}}\sum_{i=1}^{N_{B}}E_{q\left(\varepsilon \right)}\left[\log(p(y_{i}|x_{i},\mu ,\sigma ,\varepsilon ))\right] \end{array}$$

(2) Bayes back propagation algorithm

The Bayes back propagation algorithm [40,41] is used to train the proposed Bayesian deep learning model. This algorithm combines variational inference with the back propagation algorithm used in conventional deep learning model training. The object function in Equation (7) is used as an additional loss function *L*_KL_ to update the model parameters. The process of the Bayes back propagation algorithm can be expressed as follows:

Step 1: Initialize the mean and standard deviation value of random parameters as **μ** and **σ**.

Step 2: Select a batch of data from the training data.

Step 3: Generate random sample ε~N(0,I) and calculate the random parameters as W=μ+σε.

Step 4: Calculate the total loss function as *L*_total_ = *L*_mse_ + *L*_KL_, where *L*_mse_ is the mean square error function while *L*_KL_ is the KL divergence loss function as the object function in Equation (7).

Step 5: Calculate the $g_{W}^{\mathrm{mse}}$, $g_{\mu }^{\mathrm{KL}}$ and $g_{\sigma }^{\mathrm{KL}}$ which are the gradient to *L*_mse_ and *L*_KL_ by back propagation algorithm.

Step 6: Update the random parameter by gradient descent as:

$$\mu =\mu -\alpha \left(g_{W}^{\mathrm{mse}}+g_{W}^{\mathrm{KL}}+g_{\mu }^{\mathrm{KL}}\right),\;\sigma =\sigma -\alpha \left(\left(g_{W}^{\mathrm{mse}}+g_{W}^{\mathrm{KL}}\right)\varepsilon +g_{\sigma }^{\mathrm{KL}}\right)$$

Step 7: Repeat steps 2 to 6 until the deep learning model is well trained.

Where *α* is the learning rate; some adaptive gradient descent, such as Adam or RMSprop [42], can also be applied to train the proposed Bayesian deep learning model to make the model convergence in less training epochs.

### 2.3. The Framework of Proposed Method

**Figure 3:**
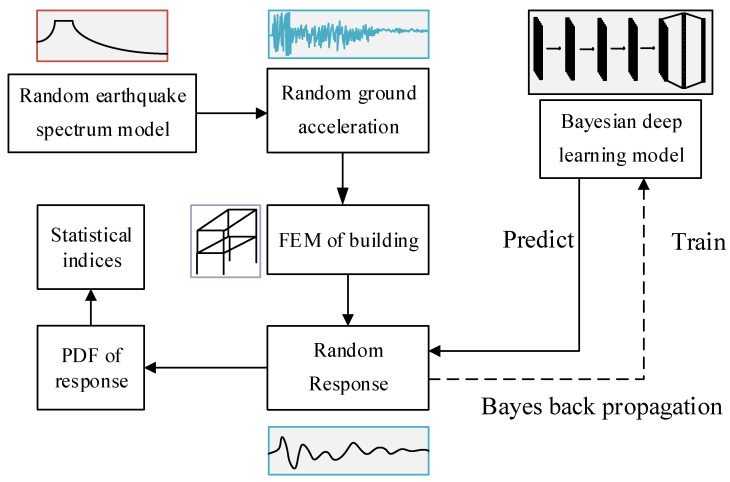
The framework of proposed method.

The framework of the proposed method is presented in Figure 3. A finite element model of a 3D building with random characteristics is built. Through Monte-Carlo simulation, samples of input ground motion acceleration and output structural response are generated. The training data are utilized to train the deep convolutional neural network through Bayes back propagation algorithm. The trained Bayesian deep learning model is used to predict the random response of the 3D building. The results are given in the form of probability distribution functions (PDFs) and the main statistical indices are expressed as mean value and standard deviation.

## 3. Three-Dimensional Random Building Structure

### 3.1. Three Dimensional Building with Random Characteristics

**Figure 4:**
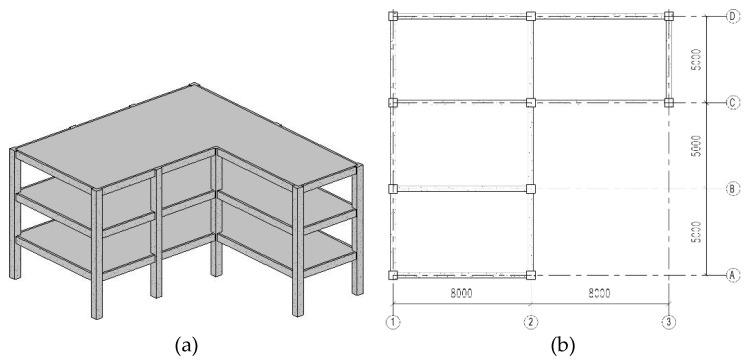
The diagram of 3D building: (**a**) 3D view; (**b**) plane view (Unit: mm).

As shown in Figure 4, a three dimensional (3D) building structure with random parameters is utilized to validate the performance of the Bayesian deep learning model. For this building, the story height for each floor is 3.0 m. The section dimension of columns and beams are 500 mm × 500 mm and 500 mm × 300 mm, respectively. The slab thickness is equal to 20 cm. In addition to the self-weight of beams and slabs, a distributed dead load of 2.5 kN/m^2^ and a live load with nominal value of 2.0 kN/m^2^ are considered in the analysis. The material parameters of concrete and steel are considered as random parameters, as shown in Table 1.

### 3.2. The Samples of Ground Motion Acceleration

In this paper, generalized the Kanai-Tajimi (GKT) [43,44,45] model is used to generate the samples of random ground motion acceleration. The GKT model can be expressed by Equations (8) and (9) as:

(8) $${\ddot{X}}_{f}\left(t\right)+2\varsigma_{g}\omega_{g}{\dot{X}}_{f}\left(t\right)+\omega_{g}^{2}X_{f}\left(t\right)=\mathrm{wn}\left(t\right),$$

(9) $${\ddot{X}}_{g}\left(t\right)=-2\left(\varsigma_{g}\omega_{g}{\dot{X}}_{f}\left(t\right)+\omega_{g}^{2}X_{f}\left(t\right)\right)e\left(t\right),$$

where **X**_f_(*t*) is the filtered response, *ω*_g_ is the characteristic ground frequency, ς_g_ is the effective ground damping coefficient, ${\ddot{X}}_{g}(t)$ is the output ground acceleration, *e*(*t*) is the amplitude envelope function, and wn(*t*) is a stationary white noise process. In this paper, the amplitude envelope function can be written as:

(10) $$E\left(t\right)=a{\left(\frac{t}{t_{n}}\right)}^{b}\exp(-c\frac{t}{t_{n}}),$$

(11) $$b=-\frac{\varepsilon_{t}\ln\eta }{1+\varepsilon_{t}\left(\ln\eta -1\right)},c=-\frac{b}{\varepsilon_{t}},a={\left(\frac{\exp(1)}{\varepsilon_{t}}\right)}^{b},$$

where *t_n_* is the duration time of the earthquake, $\varepsilon_{t}$ is normalized time when ground motion peak appears, and η is the value of ground motion at 90% of the duration time.

**Figure 5:**
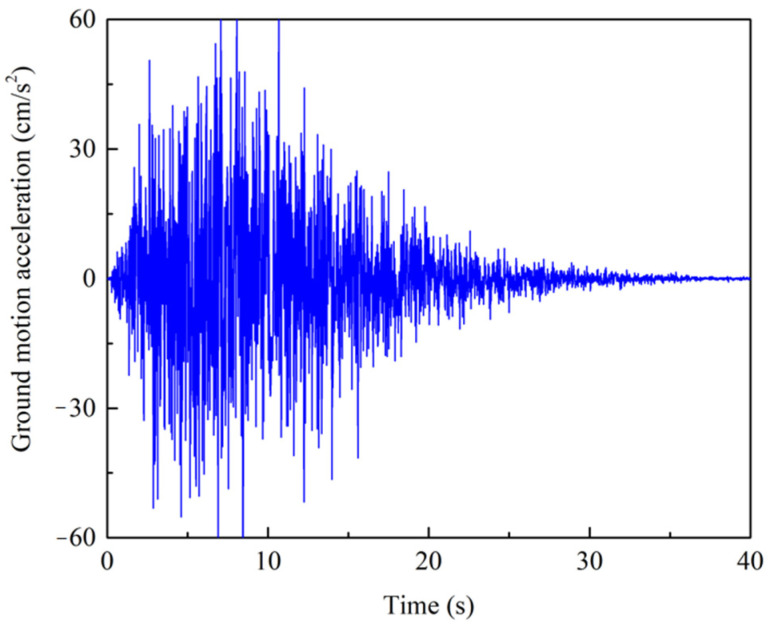
The sample of ground motion acceleration of GKT model.

The 3D building is considered to be located in a 2nd class site and the design earthquake group belongs to group 2. According to the Chinese code for seismic design of buildings and related literature [46,47], the parameters $\omega_{g}$ and $\varsigma_{g}$ are selected as *ω*_g_ = 15.7 rad/s and *ς*_g_ = 0.72. A frequent earthquake with intensity of VII is considered. Therefore, the spectral intensity of the stationary white noise process *S*_0_ is equal to 15.21 cm^2^/s^3^. The parameters of amplitude envelope function are given as: η=0.1, $\varepsilon_{t}=0.2$ and *t_n_* = 40 s. Figure 5 presents a sample generated by the GKT model.

### 3.3. Modeling and Dynamic Response Analysis of 3D Building

**Figure 6:**
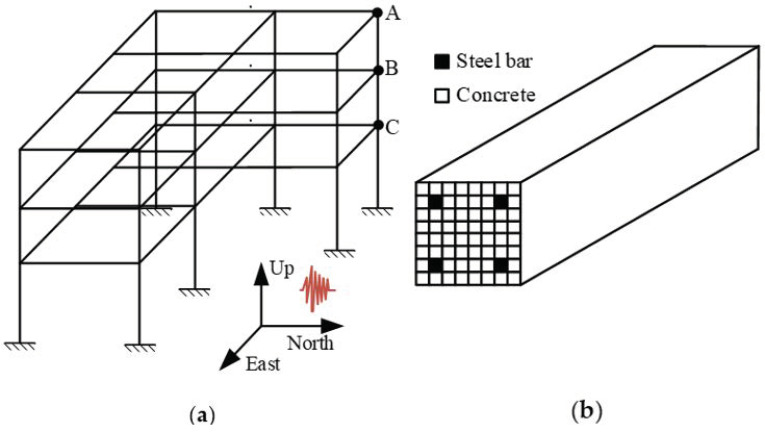
The finite element model of 3D building: (**a**) Analytic model; (**b**) Fiber approach.

**Figure 7:**
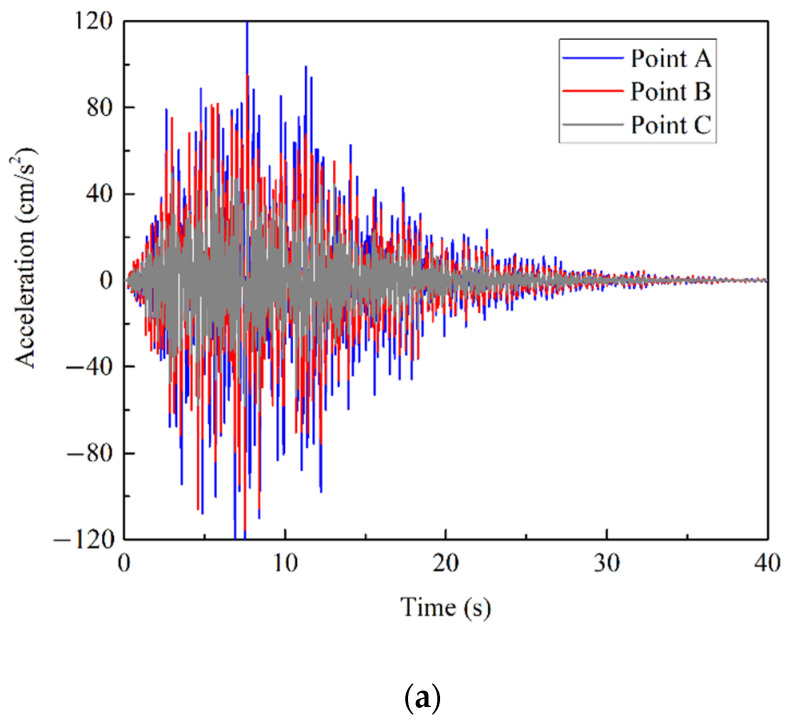

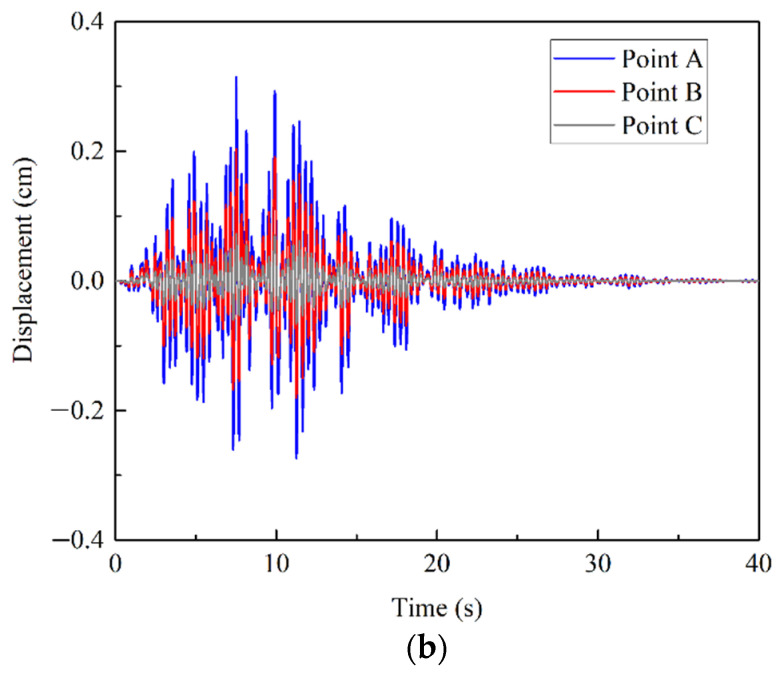
The response of 3D building under GKT ground motion: (**a**) acceleration; (**b**) displacement.

The 3D building is modeled by OpenSees [46] for further analysis. The analytic model in OpenSees can be described as in Figure 6. Every column and beam in the building is modeled through fiber approach [47]. Each fiber in the column or beam section can be assigned as a concrete or steel bar in order to simulate their combined action. The earthquake wave is applied in the direction of North to South (NS). The displacement and acceleration response of point A to C (as shown in Figure 6) is presented in Figure 7.

## 4. Numerical Results

### 4.1. Model Training

**Figure 8:**
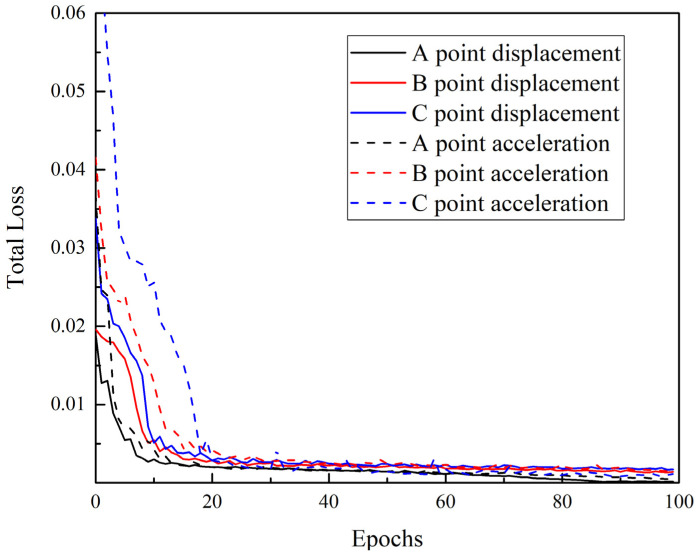
The total loss of Bayesian deep learning model varies with training epochs.

To validate the random response prediction capacity of the proposed Bayesian deep learning model, 1000 samples were generated by 3D building FE model through Monte-Carlo simulation. Out of these samples, 500 were utilized for model training while the rest were used for validation and analysis. Bayes back propagation algorithm and RMSprop algorithm were used to train the proposed Bayesian deep learning model and the initial learning rate was equal to 0.0001. The training process is presented in Figure 8 which describes the loss function, varied with training epochs. As shown, the total losses of Bayesian deep learning were reduced during modeling and after 80 epochs; the total losses arrived at the level of 10^−4^. This means the proposed model can be well trained by using the back propagation algorithm.

### 4.2. Response Prediction Results

**Figure 9:**
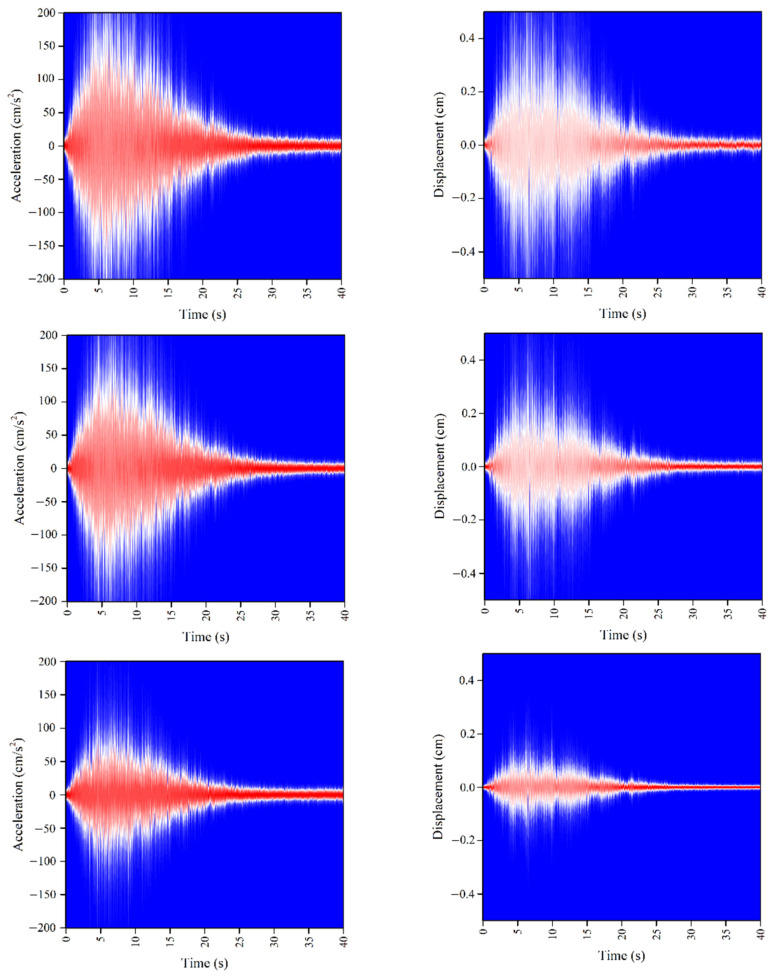

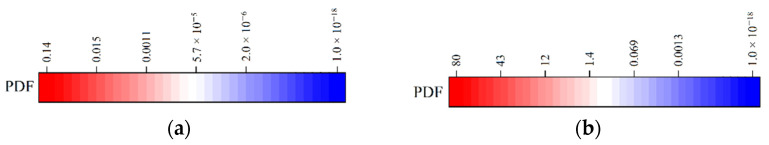
The PDF of response prediction result: (**a**) acceleration response; (**b**) displacement response. First row: point A response; Second row: point B response; point C response.

**Figure 10:**
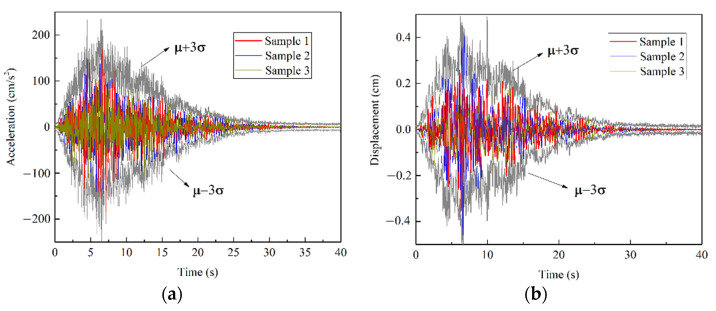
The comparison of Bayesian deep learning prediction results and results from FEM based Monte-Carlo simulation at point A: (**a**) acceleration; (**b**) displacement.

**Figure 11:**
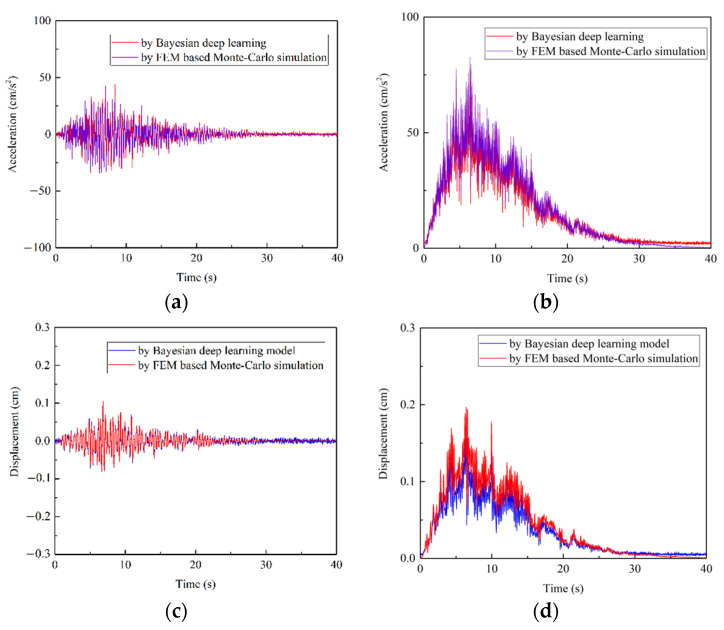
The comparison of statistical indices at point A: (**a**) acceleration mean value; (**b**) acceleration standard deviation; (**c**) displacement mean value; (**d**) displacement standard deviation.

The response prediction results are provided in the form of PDF, illustrated in Figure 9. Since the shapes of time history response curvatures at A, B and C points were similar, we only discuss the response at point A as our example. As shown in Figure 10, the samples of random response can be included in the section of [**μ** − 3**σ**, **μ** + 3**σ**], where **μ** is the mean value of response and **σ** is the standard deviation at point A. This result coincides with the conclusion of Gaussian distribution in probability theory. Figure 11 further compares the difference between Bayesian deep learning prediction results and Monte-Carlo simulation results in the mean value and standard deviation. The PDF of prediction result was close to the PDF from FEM based Monte-Carlo simulation. The normalized mean square errors (MSEs) of mean value were 1.92 × 10^−5^ (acceleration) and 5.05 × 10^−5^ (displacement), while the normalized MSEs of standard deviation were 1.17 × 10^−4^ (acceleration) and 5.10 × 10^−4^ (displacement). The peak errors of mean value were 2.56% and 5.99%, while the standard deviations were 5.34% and 10.23%. It was found that the prediction results of acceleration response had higher accuracy. This might be due to the weaker correlation between ground motion acceleration and structural displacement response.

**Figure 12:**
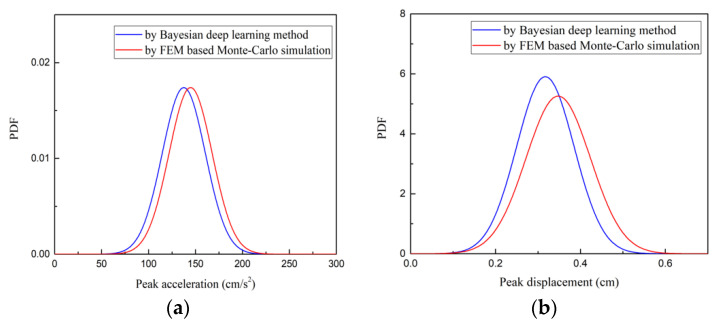
The comparison of PDF of peak value at point A: (**a**) peak acceleration; (**b**) peak displacement.

Figure 12 presents, and compares, the PDFs of peak value of acceleration and displacement responses at point A. The proposed Bayesian deep learning method can provide an effective probabilistic estimation of the peak response. The proposed method can be used as a surrogate model for seismic reliability analysis when it is combined with Monte-Carlo simulation and first passage probability theory.

## 5. Discussion

In this section, the influence of structural parameter randomness and the input noise level are discussed. The PDF of peak acceleration and peak displacement are compared, as well as their mean value and standard deviation value. Furthermore, two indices, below, are introduced as:

(12) $$Error_{\mathrm{mean}}=\frac{\left|\mathrm{Mean}\left(\mathrm{Peak}\left(X\left(t\right)\right)\right)-\mathrm{Mean}\left(\mathrm{Peak}\left(\hat{X}\left(t\right)\right)\right)\right|}{\left|\mathrm{Mean}\left(\mathrm{Peak}\left(X\left(t\right)\right)\right)\right|}$$

(13) $$Error_{\mathrm{stdv}}=\frac{\left|\mathrm{Std}(\mathrm{Peak}(X(t)))-\mathrm{Std}(\mathrm{Peak}(\hat{X}(t)))\right|}{\left|\mathrm{Std}(\mathrm{Peak}(X(t)))\right|},$$

where X(t) is the building response at point A, calculated by FEM, and $\hat{X}(t)$ is the prediction response. Peak ( ) is the peak value function, which selects the absolute maximum value from input. Mean ( ) is used to calculate the mean value of the input and Std ( ) calculates the standard deviation of the input. Equations (12) and (13) present the prediction errors of mean value and standard deviation value.

### 5.1. Influence of Parameter Randomness

**Figure 13:**
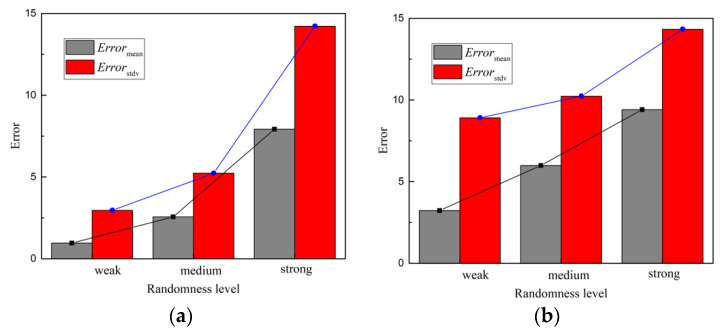
The comparison of peak error under different randomness level: (**a**) acceleration response; (**b**) displacement response.

To discuss the influence of parameter randomness, we considered three different levels of randomness. The variable coefficients of all random parameters were 0.5, 1 and 2 times the original coefficients, respectively. Slight randomness corresponds to 0.5 times variable coefficients, while 1.0 and 2.0 times correspond to medium and strong randomness. Figure 13 presents and compares the *Error*_mean_ and *Error*_stdv_ of acceleration and displacement responses at point A under different randomness levels. It can be concluded that, as the randomness of the structure parameters increases, the prediction errors increase. Due to the uncertainty simulation capacity of Bayesian deep learning, the increases in errors are considered to be limited.

### 5.2. Influence of Input Noise

**Figure 14:**
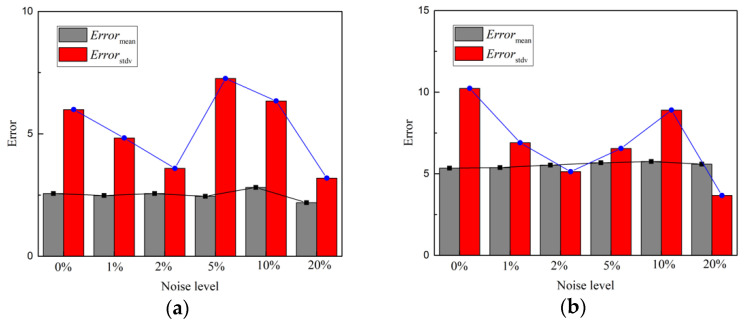
The comparison of peak errors under different noise levels: (**a**) acceleration response; (**b**) displacement response.

Robustness is the anti-noise capacity of the proposed model. In engineering practice, the input ground motion acceleration signal is polluted by noise from different sources. Therefore, robustness is very important to a deep learning model for response prediction. To discuss the influence of noise in input ground motion and the robustness of the deep learning model, ground motion data were added with 1%, 2%, 5%, 10% and 20% Gaussian white noise [48]. Figure 14 shows the Error_mean_ and Error_std_ of acceleration and displacement prediction results at point A. The mean value prediction result remained stable under different noise levels but the standard deviation results fluctuated with the noise level. However, there was no evidence that noise would influence the standard deviation prediction results, since Error_std_ does not increase with noise level. Therefore, the proposed Bayesian deep learning model is robust, even in the case of a high noise level. This is an advantage of the proposed model.

## 6. Conclusions

In this paper, a Bayesian deep learning method was proposed to predict the seismic response of a structure with random characteristics. A deep convolutional neural network, with random parameters, was established to predict the structural response. Bayes back propagation algorithm was introduced to train the deep learning model. To validate the proposed model, a 3D building structure model with random parameters was constructed in OpenSees to obtain the random response under a GKT earthquake. The Bayesian deep learning model was utilized to predict the PDF of the response. The influence of randomness of structural parameters and the noise due to ground motion were discussed. The following conclusions can be drawn:

(1) The proposed Bayesian deep learning model can reliably predict the random response of a 3D building structure with random parameters and obtain the PDF of response. The prediction of mean value is better than the prediction of standard deviation because of the parameter randomness.
(2) As the parameter randomness increases, the effective performance of Bayesian deep learning model deteriorates. Compared with mean value prediction, the standard deviation prediction is more seriously affected by parameter randomness.
(3) Input noises have very little influence on the proposed Bayesian deep learning model. The proposed model is robust and can work under a high noise level of 20%. Therefore, the proposed model has promising potential for practical applications in civil, mechanical, and aerospace engineering.

## Figures and Tables

**Table 1:** The random parameter of single floor shearing structure.

Parameter	Mean Value	Variable Coefficient	Type of Distribution
Density of concrete (kg/m^3^)	2500	0.1	Gaussian distribution
Density of steel bar (kg/m^3^)	7600	0.1	Gaussian distribution
Elastic of concrete (GPa)	30.0	0.05	Gaussian distribution
Elastic of steel bar (GPa)	210	0.05	Gaussian distribution
Damping ratio	0.05	0.05	Gaussian distribution

## Data Availability

This research received no data availability statement.
